# The relationship between bone mineral density, vitamin D level, and sleep quality in postmenopausal women with osteoporosis: a relation-seeker type study

**DOI:** 10.1590/1806-9282.20240440

**Published:** 2024-11-11

**Authors:** Emir Avsar, Selda Celik, Hande Peynirci, Feride Taskin Yilmaz, Gulden Anataca

**Affiliations:** 1 Yeditepe University, Faculty of Health Sciences, Department of Nursing – İstanbul, Turkey.; 2 University of Health Sciences, Hamidiye Faculty of Nursing – İstanbul, Turkey.; 3 University of Health Sciences, Kanuni Sultan Suleyman Training and Research Hospital, Department of Endocrinology and Metabolic Diseases – İstanbul, Turkey.; 4 Sakarya University of Applied Sciences, Faculty of Health Sciences, Department of Nursing – Sakarya, Turkey.; 5 University of Health Sciences, Kanuni Sultan Suleyman Training and Research Hospital, Department of Diabetes Nursing – İstanbul, Turkey.

**Keywords:** Osteoporosis, Bone density, Vitamin D, Sleep quality

## Abstract

**OBJECTIVE:**

This study was conducted to determine the relationship between bone mineral density, vitamin D level, and sleep quality in female patients with osteoporosis.

**METHODS:**

This descriptive correlational study included a total of 318 women diagnosed with osteoporosis. The data were collected using a patient identification form, including items for the plasma vitamin D level and bone mineral density score obtained by the dual-energy X-ray absorptiometry method, and the Pittsburgh Sleep Quality Index.

**RESULTS:**

The mean age of the women was 56.49±5.68 years, and their femoral neck T mean score, an indicator of bone mineral density, was −2.94±0.31. Only 6.3% of the women had adequate vitamin D levels. In addition, according to their Pittsburgh Sleep Quality Index scores, 85.8% of the women had poor sleep quality. In the study, no significant difference was found between the women's bone mineral densities and vitamin D levels according to sleep quality (p>0.05). However, there was a weak negative correlation between the duration of osteoporosis, body mass index, and sleep quality (p<0.05).

**CONCLUSION:**

There was no association between the vitamin D level, bone mineral density, and sleep quality, but the duration of osteoporosis was negatively associated with sleep quality. Accordingly, it may be recommended to provide education and counseling to postmenopausal women diagnosed with osteoporosis on issues such as sunbathing, vitamin D and calcium preparation intake, weight control, and non-pharmacological treatment approaches by making necessary individual-specific plans to improve sleep quality.

## INTRODUCTION

Osteoporosis is an important public health problem characterized by loss of bone mass and deterioration of the microstructure of bone tissue, with a high prevalence associated with increased mortality and mobility^1^. Osteoporosis is three times more likely to occur in women than in men, and it is an essential aspect of women's health during aging^2,3^. In 2020, a meta-analysis of 70 studies involving a total of 800,457 women found that the worldwide prevalence of osteoporosis in women was 23.1%^4^. The FRACTURK study conducted by the Osteoporosis Association of Turkey reported the prevalence of osteoporosis in women aged 50 years and older in Turkey as 12.9%, and this rate increased to 37.7% in women aged 80 years and older^5^.

While gender, age, ethnicity, and genetic characteristics are non-modifiable risk factors for the development of osteoporosis, obesity, inadequate calcium intake, and low vitamin D levels are among the modifiable risk factors^6,7^. In addition, some studies have reported that poor sleep quality is among the factors that increase the risk of osteoporosis^8,9^. There are few studies examining the relationship between bone mineral density and sleep quality^10–13^. Some of these studies found that sleep quality decreased as bone mineral density decreased^10,11^, while some studies determined no significant relationship between bone mineral density and sleep quality^14,15^.

Although there are studies examining the relationship between vitamin D levels and sleep quality^15–19^, the results are not consistent. In addition to studies showing that vitamin D has a significant positive effect on sleep quality^15–17^, there are also studies showing that vitamin D has no significant effect on sleep quality^18,19^. This study was needed because studies on the relationship of bone mineral density and vitamin D levels with sleep quality in the literature yielded conflicting results.

The research questions of our study for postmenopausal women with osteoporosis are as follows:

1. Is there a relationship between bone mineral density and sleep quality?
2. Is there a relationship between vitamin D levels and sleep quality?
3. Is there a relationship between age, duration of osteoporosis, body mass index (BMI), non-pharmacological methods used for sleep, and sleep quality?

This study was conducted to determine the relationship between bone mineral density, vitamin D level, and sleep quality in postmenopausal female patients with osteoporosis.

## METHODS

The population of this descriptive correlational study consisted of female patients who applied to the internal medicine and endocrinology and metabolism diseases outpatient clinics of a training and research hospital in Istanbul for diagnosis/treatment/follow-up between July and October 2022. A total of 401 patient files were reviewed for our study. To determine the sample size, a power analysis was conducted using the G Power 3.1 software. According to the results of this analysis, with an effect size of 0.50, a power of 95%, and an error margin of 0.5, the minimum required sample size was calculated to be 230. Considering the potential for a 20% loss during data collection, the sample size was planned to be at least 250 participants. In this context, a total of 318 female patients diagnosed with osteoporosis who met the inclusion criteria constituted the sample of the study. Study inclusion criteria were as follows: being over 18 years of age, being a female patient diagnosed with osteoporosis, being in the postmenopausal period, being able to communicate verbally, and agreeing to participate in the study. Study exclusion criteria were as follows: having a malignant tumor with bone metastasis, having thyroid disease, having a diagnosis of pituitary disease, using chronic regular steroids for more than 6 months, having a diagnosis of psychiatric disease, missing file data (vitamin D value, dual-energy X-ray absorptiometry—DXA—result), and taking medication that causes sleep problems. A total of 83 patients were excluded from the study because 58 patients had incomplete file data (22 due to vitamin D values and 36 due to DXA results), 12 patients were followed up with a diagnosis of psychiatric disease, 10 patients were taking medication for sleep problems, and 3 patients refused to participate in the study.

The data were collected using a "patient identification form" and the "Pittsburgh sleep quality index."

### Patient identification form

This form was prepared by the researchers as a result of the literature review and consisted of two different sections, "sociodemographic characteristics" and "characteristics related to the disease and sleep quality," and 16 questions in total^7,10,11^. The patient's weight and height values were obtained from the last measurements, and vitamin D values and bone mineral density (femoral neck T-scores) were retrieved from patient files that included the results of the last laboratory tests.

### Pittsburgh Sleep Quality Index (PSQI)

This scale was developed by Buysse et al.^20^ in 1989 to assess sleep quality, and its Turkish validity and reliability study was conducted by Agargun et al. This scale consists of 19 items and 7 subscales. The seven subscale scores are then summed to yield a total PSQI score, which ranges between 0 and 21. Those with a total score of ≤5 and below are considered to have "good sleep quality," and those with a score of >5 are considered to have "poor sleep quality"^21^. In this study, Cronbach's alpha internal consistency coefficient of the scale was determined as 0.76.

### Data collection

The data were collected by the researchers through face-to-face interviews with patients who were referred to our polyclinic, and the average interview time for each individual was 15 min. Parameters such as BMI, plasma vitamin D level, measurement of bone mineral density, and diagnosis of osteoporosis were obtained by considering certain standards. According to the National Institute of Health and the World Health Organization (WHO), BMI≤18.5 kg/m^2^ is defined as underweight, between 18.5 and 24.9 kg/m^2^ as normal weight, between 25 and 29.9 kg/m^2^ as overweight, and BMI≥30 kg/m^2^ as obese^22^. Therefore, in this study, when individuals applied to the outpatient clinic in the morning on an empty stomach for examination, their weight and height were measured and their BMI values were calculated and categorized in accordance with the abovementioned criteria. In accordance with the physician's request for patients who applied to the outpatient clinic for follow-up, their vitamin D results were obtained from the patient file with the Elecsys Vitamin D Total III kit, which was studied by the electrochemiluminescence method on the Cobas e801 device. To ensure standardization in the study, a categorization was made considering the reference range published by the Turkish Society of Endocrinology and Metabolism (TSEM) based on Turkish data. According to the TSEM, a 25-OH vitamin D level of 20 ng/mL and above is defined as normal/adequate, between 10 and 20 ng/mL as vitamin D insufficiency, and below 10 ng/mL as vitamin D deficiency^23^. The diagnosis of osteoporosis is based on the WHO's diagnostic criteria. Considering these criteria in terms of bone mineral density values according to the young adult average; T-score≥-1.0 is classified as normal, −1>T-score>-2.5 as osteopenia (low bone mass), T-score≤-2.5 as osteoporosis, and T-score≤−2.5 with one or more osteoporosis-related fractures as severe osteoporosis (established osteoporosis)^24^. Since all female patients were in the postmenopausal period, T-score was used in the study, and those with T-score≤-2.5 were included in the sample selection. The measurement of bone mineral density was conducted as part of a routine follow-up under the physician's request. No additional radiological assessments were performed for the study.

### Data analysis

The data were analyzed using the I.B.M SPSS (Statistical Package for Social Sciences) 22.0 package program. The participants’ sociodemographic characteristics, disease parameters, bone health parameters, and scale mean scores were evaluated using percentage and mean tests. The chi-square test was employed to examine the relationship between vitamin D levels and sleep quality. Additionally, Student's t-test and Pearson's correlation test were used to determine the relationship between certain characteristics of the participants and their scale mean scores. The results were evaluated at a 95% confidence interval, and the significance level was set at p<0.05.

### Ethical considerations

For conducting the study, approval was obtained from the Clinical Research Ethics Committee of the relevant institution (Number: 2022.07.169, Subject No: KAEK/2022.07.169), and written permission (Number: E-l59l6306-604.01.0l) was obtained from the relevant institution where the research would be conducted and the data would be collected. In addition, before data collection, each patient who met the inclusion criteria and agreed to participate in the study was informed about the purpose and content of the study and their verbal and written consent was obtained.

## RESULTS

The records of 401 potentially eligible patients were reviewed. Upon evaluation against the eligibility criteria, 318 patients were included in the study. Relevant questionnaires were administered; necessary data were obtained from their records, and the data were analyzed using appropriate methods.

The mean age of the women was 56.49±5.68 years; 65.7% of them were primary school/middle school graduates, 22.6% were high school graduates, 11.7% were illiterate, and 6.3% were employed. In addition, 10.1% of the women were smokers and all of them did not consume alcohol. Furthermore, 26.4% of the women had chronic diseases other than osteoporosis, where 67.9% of them had diabetes, 26.2% had hypertension, and 10.7% had coronary artery disease. In addition, 42.5% of the women resorted to non-pharmacological methods such as the consumption of herbal tea to improve their sleep quality. The women's femoral neck T mean score, an indicator of bone mineral density, was −2.94±0.31, and only 6.3% of the women had adequate vitamin D levels. In addition, according to the women's PSQI scores, 85.8% of them had poor sleep quality (Table 1).

**Table 1:** Distribution of patients’ characteristics regarding osteoporosis, risk factors, and Pittsburgh Sleep Quality Index scores (n=318).

Features		n	%
Osteoporosis duration (years) (mean±SD) (min–max)		1.82±0.73 (1–4)	
BMI (kg/m²) (mean±SD) (min–max)		24.62±3.04 (19.10–38.50)	
BMI category			
	18.5–24.9 kg/m²	206	64.8
	25.0–29.9 kg/m²	96	30.2
	≥30 kg/m²	16	5.0
Presence of a family member with osteoporosis			
	Yes	153	48.1
	No	165	51.9
History of fractures resulting from a fall, crash, blow in the past			
	Yes	127	39.9
	No	191	60.1
Presence of a family member with a fracture history of fall, crash, blow in the past			
	Yes	135	42.5
	No	183	57.5
Sun exposure			
	Over 1 month per year	50	15.7
	Less than 1 month per year	154	48.5
	Never	114	35.8
Calcium supplementation			
	Yes	196	61.6
	No	122	38.4
Vitamin D supplementation			
	Yes	109	34.3
	No	209	65.7
Exercise status			
	3–4 times a week for 30 min	13	4.1
	1–2 times a week for 30 min	36	11.3
	Irregular	111	34.9
	Never	158	49.7
Coffee consumption habit			
	Yes	193	60.7
	No	125	39.3
Vitamin D level categories			
	Sufficient vitamin D	20	6.3
	Insufficiency of vitamin D	227	71.4
	Deficiency of vitamin D	71	22.3
Sleep quality (PSQI scores)			
	Good	45	14.2
	Bad	273	85.8
Parameters		Mean±SD	
Bone mineral density (femoral neck T-scores)		-2.94±0.31	
25 (OH) vitamin D level (ng/mL)		13.80±4.22	

BMI: body mass index; PSQI: Pittsburgh Sleep Quality Index; SD: standard deviation.

The study found no significant difference between the women's bone mineral densities and vitamin D levels according to sleep quality (p>0.05) (Table 2).

**Table 2:** Comparison of patients’ bone mineral density and vitamin D levels with Pittsburgh Sleep Quality Index average score.

Features	Sleep quality		Test; p
	Good (n=45)	Bad (n=273)	
	Mean±SD	Mean±SD	
Bone mineral density	-2.90±0.33	-2.94±0.31	t=0.927; 0.354
25 (OH) vitamin D level	14.28±3.87	13.72±4.28	t=0.834; 0.405
	**n (%)**	**n (%)**	
Vitamin D level categories			
Sufficient vitamin D	3 (6.7)	17 (6.2)	χ^2^=1.389; 0.499
Insufficiency of vitamin D	35 (77.8)	192 (70.3)	
Deficiency of vitamin D	7 (15.6)	64 (23.4)	

This study found a weak negative correlation between the women's duration of osteoporosis and sleep quality (p<0.05). The overweight–obese women had worse sleep quality than those with normal weight (p<0.05). The women who used non-pharmaceutical methods for sleep (herbal tea) had significantly better sleep quality (p=0.000). However, the variables of taking calcium and vitamin D supplements and having chronic diseases other than osteoporosis did not show any difference in terms of sleep quality (p>0.05) (Table 3).

**Table 3:** Comparison of some characteristics of patients and Pittsburgh Sleep Quality Index average score.

Features		Pittsburgh Sleep Quality Index	Test; p
		Mean±SD	
Age (years)			r=-0.126; **p=0.025**
Osteoporosis duration (years)			r=-0.142; **p=0.011**
Body structure according to BMI			
	18.5–24.9 kg/m² arasında		
	25.0–29.9 kg/m² arasında		
	30 kg/m² ve üzerinde		
Normal weight		10.29±3.58	t=-2.460; **p=0.011**
Overweight–obese		11.27±3.02	
Calcium supplementation			
	Yes	10.81±3.38	t=1.155; p=0.249
	No	10.36±3.48	
Vitamin D supplementation			
	Yes	10.43±3.42	t=0.791; p=0.430
	No	10.75±3.42	
Using any nondrug method for sleep			
	Yes	9.57±3.78	t=-6.943; **p=0.000**
	No	12.08±2.13	
Presence of another chronic disease			
	Yes	10.28±3.76	t=-1.111; p=0.267
	No	10.76±3.29	

Significant p-values (p<0.05) are indicated in bold. BMI: body mass index; SD: standard deviation.

## DISCUSSION

Osteoporosis causes many physical disorders and may lead to sleep problems in individuals^24^. In studies conducted with individuals diagnosed with osteoporosis, the prevalence of poor sleep quality was found to range between 50 and 74.77%^25–27^. In this study, although those with good sleep quality had higher bone mineral density compared to those with poor sleep quality, the difference between them was not statistically significant. Another study conducted in Turkey found no significant relationship between the femoral neck T-score and sleep quality in postmenopausal women with osteoporosis^13^. In contrast to these studies, a study by Lin et al. involving 2,067 Chinese women found a positive correlation between bone mineral density and sleep quality^15^. The reason why the result of our study differs from those in some studies in the literature may be due to the presence of different ethnic groups and therefore the effect of genetic differences and the use of different measurement tools in the evaluation of sleep, taking bone mineral densities in different body parts and the different living conditions of individuals.

Many diseases are known to reduce bone mineral density, which can be managed through technology-based treatments, conventional therapies, and vitamin supplements^28,29^. Vitamin D deficiency negatively impacts hormones related to bones and muscles^30^. In this study, the rate of postmenopausal women with vitamin D insufficiency/deficiency was 93.7%, and there was no significant relationship between the women's plasma vitamin D level, status of vitamin D supplement use, and sleep quality. Mason et al. found that vitamin D supplementation given to postmenopausal women for 12 months did not significantly change their sleep quality^19^. Therefore, our study data support those in the literature.

Sasaki et al. examined the effect of sleep quality on osteoporosis and found that BMI had a significant effect on sleep quality^31^. In another study conducted in China with 247 patients diagnosed with osteoporosis, overweight–obese individuals had worse sleep quality than normal weight and lean individuals^32^. In the present study, overweight–obese women were found to have worse sleep quality than normal-weight women. Therefore, our study data are in line with those in the literature.

Herbal teas and herbal preparations are among the methods frequently preferred by individuals with poor sleep quality. There is no study evaluating the effect of herbal teas/products on sleep quality in female patients with osteoporosis. Our study found that sleep quality was higher in women with osteoporosis who consumed herbal tea.

### Limitations of the study

The study was conducted in a single center and in a specific time period. Since only female patients diagnosed with osteoporosis who met the inclusion criteria and agreed to participate in the study participated in the study, the study results cannot be generalized to all women with osteoporosis.

## CONCLUSION

This study determined that the majority of women diagnosed with osteoporosis had poor sleep quality; however, sleep quality was negatively associated with the duration of osteoporosis and BMI and positively associated with herbal tea consumption. Accordingly, it is recommended that health professionals should be sensitive about sleep quality in women with osteoporosis, make necessary individual-specific plans to improve their sleep quality, and provide them with education and counseling on issues such as sunbathing status, vitamin D, and calcium preparation intake and non-pharmacological treatment approaches.
